# Spatial Characteristics of Life Expectancy and Geographical Detection of Its Influencing Factors in China

**DOI:** 10.3390/ijerph17030906

**Published:** 2020-02-01

**Authors:** Yafei Wu, Ke Hu, Yaofeng Han, Qilin Sheng, Ya Fang

**Affiliations:** 1 State Key Laboratory of Molecular Vaccine and Molecular Diagnostics, School of Public Health, Xiamen University, Xiamen 361102, China; wyfyyahcx@163.com (Y.W.); huke2296991140@163.com (K.H.); yaofenghan@163.com (Y.H.); shengqilin5@163.com (Q.S.); 2 Key Laboratory of Health Technology Assessment of Fujian Province, School of Public Health, Xiamen University, Xiamen 361102, China; 3 National Institute for Data Science in Health and Medicine, Xiamen University, Xiamen 361102, China

**Keywords:** life expectancy, spatial characteristics, social and environmental factors, spatial stratified heterogeneity, Geographical Detector

## Abstract

Life expectancy (LE) is a comprehensive and important index for measuring population health. Research on LE and its influencing factors is helpful for health improvement. Previous studies have neither considered the spatial stratified heterogeneity of LE nor explored the interactions between its influencing factors. Our study was based on the latest available LE and social and environmental factors data of 31 provinces in 2010 in China. Descriptive and spatial autocorrelation analyses were performed to explore the spatial characteristics of LE. Furthermore, the Geographical Detector (GeoDetector) technique was used to reveal the impact of social and environmental factors and their interactions on LE as well as their optimal range for the maximum LE level. The results show that there existed obvious spatial stratified heterogeneity of LE, and LE mainly presented two clustering types (high–high and low–low) with positive autocorrelation. The results of GeoDetector showed that the number of college students per 100,000 persons (NOCS) could mainly explained the spatial stratified heterogeneity of LE (Power of Determinant (*PD*) = 0.89, *p* < 0.001). With the discretization of social and environmental factors, we found that LE reached the highest level with birth rate, total dependency ratio, number of residents per household and water resource per capita at their minimum range; conversely, LE reached the highest level with consumption level, GDP per capita, number of college students per 100,000 persons, medical care expenditure and urbanization rate at their maximum range. In addition, the interaction of any two factors on LE was stronger than the effect of a single factor. Our study suggests that there existed obvious spatial stratified heterogeneity of LE in China, which could mainly be explained by NOCS.

## 1. Introduction

Life expectancy (LE) is a comprehensive and important index for measuring population health, which is vital for policy development and health improvement [1]. In recent years, the LE has continued to rise with the development of economy and living standard in China. From 2000 to 2017, it increased from 71.40 to 76.47 years old [2]. However, LE showed obvious spatial differences in China. According to a research, the difference of LE was larger than 10 years between the east and west in China in 2010 [3]. Therefore, it is an urgent task to explore the spatial characteristics and influencing factors of LE in order to deal with the regional inequality of LE in China.

There have been many studies concerning the various influencing factors of LE. These factors can mainly be divided into two categories: biological and social and environmental factors [4]. Biological factors involve the individual’s genetic factors, living habits, etc. Differences in LE between men and women are mainly due to differences in gene expression intensity. In addition, a study also found that people who smoked, drank and exercised less were vulnerable to cancer, cardiovascular and cerebrovascular diseases, which caused a reduction of LE [5]. However, social and environmental factors were the most frequently considered and important influencing factors in LE research [6]. Many studies found that economy and demographic composition were key influencing factors of LE [7,8,9,10]. Studies have found that health care and services had a positive effect on disease prevention and treatment, so the LE level was often higher in areas with good medical conditions [11]. People with a higher education level tend to have better health awareness, so the LE of these individuals is higher. The emergence of education differences would enlarge the gap of LE among different populations [12]. Moreover, researchers also found that other social and environmental factors, such as environmental resources, had some impact on LE [13]. However, these studies were mainly based on few social and environmental factors, and they rarely explored the effects of multi-dimensional factors and their interactions from a spatial perspective.

Multiple linear regression (MLR) is the most widely used method for traditional studies of LE [14], while MLR has not yet considered the spatial information contained in data and can not effectively deal with multiple collinearity and interactions between variables. Furthermore, global and local spatial regression models, such as Spatial Error Model (SEM), Spatial Lag Model (SLM) and Geographical Weighted Regression (GWR), have been applied to LE research [15]. Although the above spatial methods can deal with the spatial autocorrelation and heterogeneity of LE and independent variables, the spatial stratified heterogeneity of LE have not been explored yet. As a result, the Geographical Detector technique has been put forward and developed rapidly in recent years. The geographical detector is a specific tool developed by Wang for spatial stratified heterogeneity study without any assumptions on the distribution of dependent and independent variables [16,17]. It can detect the spatial stratified heterogeneity of dependent variables and reveal the driving force behind it with its great flexibility [18]. It has been widely used in various fields, such as natural and social sciences [19,20,21], environmental pollution [22,23] and disease risk detection [16,24], etc., but it is rarely used in the field of LE research.

Therefore, we analyzed the spatial distribution characteristics of LE by descriptive methods and spatial autocorrelation analysis. Then we used the Geographical Detector technique to reveal the impact of social and environmental factors and their interactions on LE as well as their optimal range for the maximum LE level, providing reference for the research on LE, economic and educational development, utilization of medical resources, environmental protection as well as population management policy-making in China.

## 2. Materials and Methods

### 2.1. Data

In order to explore the impact of social and environmental factors on LE in China, we selected 11 representative social and environmental factors (Table 1) classified to 7 categories. We performed an analysis at the provincial level, including 31 provinces in mainland China. The LE data in 2010 came from China Statistical Yearbooks [25]. The data of TDR (Total dependency ratio) in 2010 was obtained from China Population & Employment Statistics Yearbooks [26], and the data of MCEOUR (Medical care expenditure of urban residents) in 2010 came from China Health Statistics Yearbooks [27]. The data of other social and environmental factors in 2010 were all from China Statistics Yearbooks [28,29].

### 2.2. Statistical Methods

#### 2.2.1. Descriptive Methods

In order to describe the spatial distribution of LE and social and environmental factors, the LE and main social and environmental factors of each province were mapped (the value was represented by the color depth) based on the provincial scale map of mainland China.

#### 2.2.2. Spatial Autocorrelation Analysis

In order to analyze the spatial clustering characteristics of LE in China, we conducted the global and local spatial autocorrelation by using Moran’s index (Moran’s I).

##### Global Spatial Autocorrelation Analysis

Global Moran’s I is used to judge whether there exists spatial autocorrelation in LE in the whole area. Moran’s I ranges from −1 to 1. If it is greater than 0, spatial autocorrelation exists in LE; if it equals to 0, no spatial autocorrelation exists in LE, if it is less than 0, spatial discreteness exists in LE. Global Moran’s I can be expressed as follows [30]:

(1) $$I=\frac{n\sum_{i=1}^{n}\sum_{j=1}^{n}W_{ij}(x_{i}-\bar{x})(x_{j}-\bar{x})}{\sum_{i=1}^{n}\sum_{j=1}^{n}W_{ij}\sum_{i=1}^{n}{\left(x_{i}-\bar{x}\right)}^{2}}$$

where *n* represents the number of spatial units (provincial administrative unit in the study); *x_i_* and *x_j_* is the LE of the *i* and *j* province, respectively; $\bar{x}$ is the average of the LE, and $W_{ij}$ is the spatial weight matrix based on the inverse distance as follows:

(2) $$W_{ij}={d_{ij}}^{-\alpha }$$

where *d_ij_* refers to the distance between two provinces, *α* is the appropriate constant (such as 1 or 2).

##### Local Spatial Autocorrelation Analysis

We used local Moran’s I (*LISA*) to detect the spatial cluster types of LE further. And there are 4 types: high–high (high value is surrounded by high value), low–low (low value is surrounded by low value), high–low (high value is surrounded by low value) and low–high (low value is surrounded by high value) [31]. The local Moran’s I is calculated as follows [32]:

(3) $$Ii=\frac{n(x_{i}-\bar{x})\sum_{j=1}^{n}W_{ij}(n_{j}-\bar{n})}{\sum_{j=1}^{n}{\left(x_{j}-\bar{x}\right)}^{2}}$$

Each index in the above formula is the same as Equation (1). In addition, we also showed the spatial cluster type of LE by creating a Lisa cluster map.

#### 2.2.3. Geographical Detector

Based on the spatial variance analysis, Geographic detector is a new tool to detect environmental factors of health risk. By comparing between strata-variance with the total variance in the whole area of dependent variable, the Geographical Detector can detect whether the factor causes the spatial stratified heterogeneity of dependent variable or not [24]. The Geographical Detector consists of 4 parts: factor detector, ecological detector, risk detector and interaction detector. Each of its components is described in detail as follows:

##### Factor Detector

A factor detector can be used to detect the importance of certain factors on LE, and is commonly measured by the Power of Determinant (*PD*) as follows [33]:

(4) $$PD=1-\frac{\sum_{h=1}^{L}N_{h}\sigma_{h}^{2}}{N\sigma^{2}}$$

where *h* = 1,…,L refers to a certain stratum of each factor (L is the number of strata of the factor); $\sigma^{2}$ and $\sigma_{h}^{2}$ represents the variance of LE in the whole and stratum *h*, respectively, *N* and *N_h_* represent the sample for them, respectively. *PD* ranges from 0 to 1. If *PD* is closer to 1, the effect of this factor on LE is greater. And the *p*-value of *PD* was also given by the factor detector.

##### Risk Detector

The risk detector is used to judge whether there is a significant difference between the average LE of different strata of each factor. *T*-test is used for hypothesis tests [16]:

(5) $$t_{{\bar{y}}_{h-1}-{\bar{y}}_{h-2}}=\frac{{\bar{Y}}_{h=1}-{\bar{Y}}_{h=2}}{{\left[\frac{\mathrm{Var}\left({\bar{Y}}_{h=1}\right)}{n_{h=1}}+\frac{\mathrm{Var}\left({\bar{Y}}_{h=2}\right)}{n_{h=2}}\right]}^{1/2}}$$

The degree of freedom was:

(6) $$df=\frac{\frac{\mathrm{Var}\left({\bar{Y}}_{h=1}\right)}{n_{z=1}}+\frac{\mathrm{Var}\left({\bar{Y}}_{h=2}\right)}{n_{h=2}}}{\frac{1}{n_{h=1}-1}{\left[\frac{\mathrm{Var}\left({\bar{Y}}_{h=1}\right)}{n_{h=1}}\right]}^{2}+\frac{1}{n_{h=2}-1}{\left[\frac{\mathrm{Var}\left({\bar{Y}}_{h=2}\right)}{n_{h=2}}\right]}^{2}}$$

where $\bar{Y}h$ represents the average LE of the stratum *h*; *n_h_* indicates samples for stratum *h*; *Var* represents sample variance, *t* follows the Student’s-*t*-test distribution. The null hypothesis refers to the equivalent of LE in two areas. If null hypothesis is rejected at the significance level α, it is considered that there exists a significant difference in LE between the two areas.

##### Ecological Detector

The ecological detector is able to detect whether there is a significant difference between the effects of two factors on LE. And it is measured by the following formula:

(7) $$\begin{array}{c} F=\frac{n_{X1}\left(n_{x2}-1\right)SSW_{X1}}{n_{X2}\left(n_{x1}-1\right)SSW_{X2}}\\ SSW_{X1}=\sum_{h=1}^{L1}N_{h}\sigma_{h}^{2}\;\;SSW_{X2}=\sum_{h=1}^{L2}N_{h}\sigma_{h}^{2} \end{array}$$

where $n_{x1}$ and $n_{x2}$ represent the samples; *SSWX*_1_ and *SSWX*_2_ represent the sum of within-strata variance of strata divided by *x*_1_ and *x*_2_ (any two social and environmental factors in the study) respectively; *L*_1_ and *L*_2_ represent the number of strata of variables *x*_1_ and *x*_2_ respectively; The null hypothesis represent *SSWX*_1_ and *SSWX*_2_ are equal. If null hypothesis is rejected at the significance level of α, this indicates that there is a significant difference between the effects of two factors on LE.

##### Interaction Detector

The interaction detector is used to identify the interaction between different factors. It can evaluate whether the interaction of factors *x*_1_ and *x*_2_ will increase or decrease the influence on LE. Specifically, it calculates the *PD* value of *x*_1_ and *x*_2_ after interaction ($X_{1}\cap X_{2}$) and then compares it with *PD*(*x*_1_) and *PD*(*x*_2_) to judge the interaction type. As shown in line 3 of Table 2, the *PD* value of *x*_1_ and *x*_2_ after interaction is greater than the maximum of the original *PD* values of the two factors, suggesting that the interaction type is bivariate-enhanced.

### 2.3. Data Preprocessing

The Geographical Detector analysis generally requires the independent variable to be categorical variable, so it is necessary to discretize the 11 social and environmental factors before the analysis. Different discretization schemes will have different impact on performance. Generally, the discretization scheme with the largest *PD* value is preferred in a geographical detector analysis [35]. After comparison of various discretization schemes, we used the quantile method to classify the above 11 social and environmental variables and the classification interval is shown in Table 3. As for the dependent variable, it can be either continuous or discrete, so we did not make any preprocessing.

### 2.4. Software

The discretization of social and environmental factors and the spatial autocorrelation analysis were implemented by ArcGIS 10.2, and the analysis of spatial stratified heterogeneity of LE was completed by the GeoDetector.

## 3. Results

### 3.1. Spatial Distribution Characteristics of LE in China

**Figure 1:**
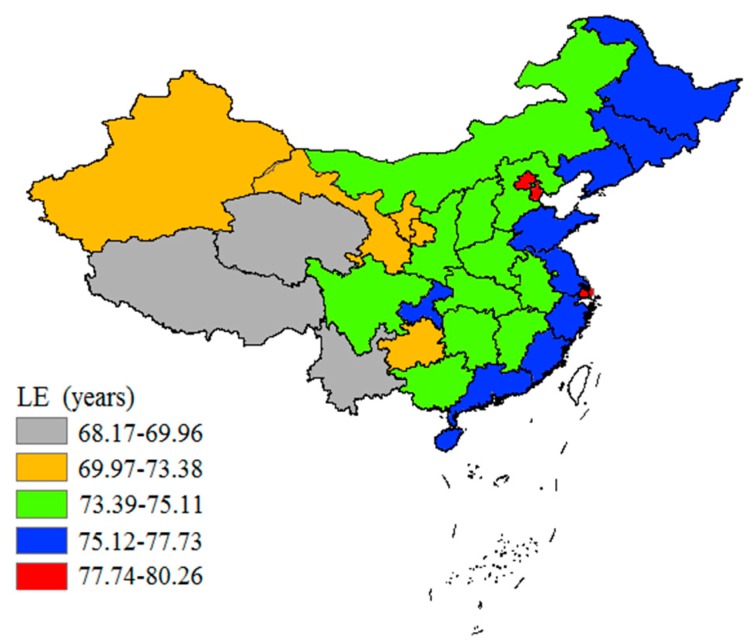
Distribution of LE (Life expectancy) in China in 2010.

In 2010, the average LE was 74.83 years old in China, and the average LE of women was higher than that of men (77.37 vs. 72.38). The results show that LE showed a clear downward trend from the east to the west in 2010 in China, which reveals that there existed obvious spatial stratified heterogeneity of LE in China (Figure 1). The average LE of the eastern areas was over 75 years old, especially in Shanghai and Beijing. The average LE of the central areas was a bit lower than the eastern areas which was between 73.39 and 75.11 years, while the average LE in the western areas was the lowest in China. Tibet had the lowest LE of 68.17 years in all the 31 provinces.

**Figure 2:**
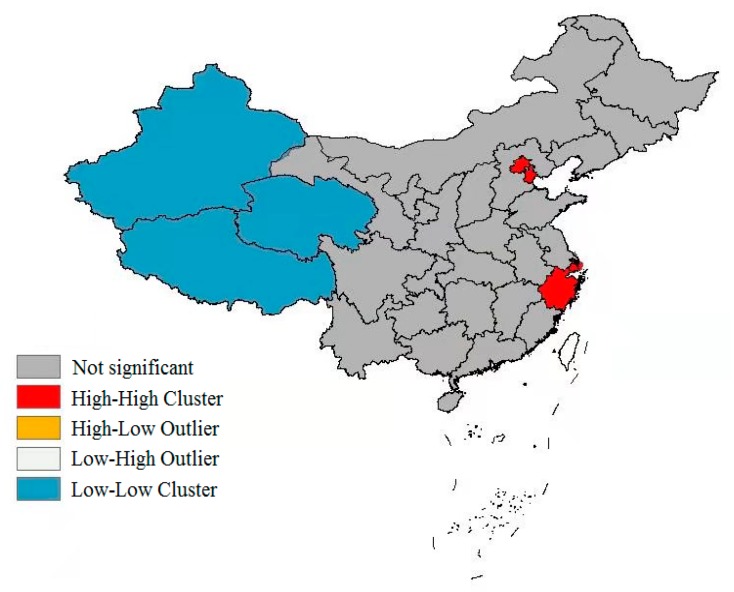
Lisa cluster map of LE in China in 2010.

From the results of the global autocorrelation analysis, the global Moran’s I was 0.266 (*p* < 0.001), indicating that there existed obvious spatial clustering for LE in China. In order to further explore the cluster type of LE, we conducted a local autocorrelation analysis and obtained a Lisa cluster map (Figure 2). It can be seen from Figure 2 that LE mainly presented two cluster types: high–high and low–low, among which high–high types were mainly distributed in the eastern coastal areas, including Beijing, Tianjin, Shanghai and Zhejiang provinces. The low–low type was mainly located in Western inland regions, such as Xinjiang, Tibet and Qinghai provinces.

### 3.2. Spatial Distribution of Main Social and Environmental Factors

**Figure 3:**
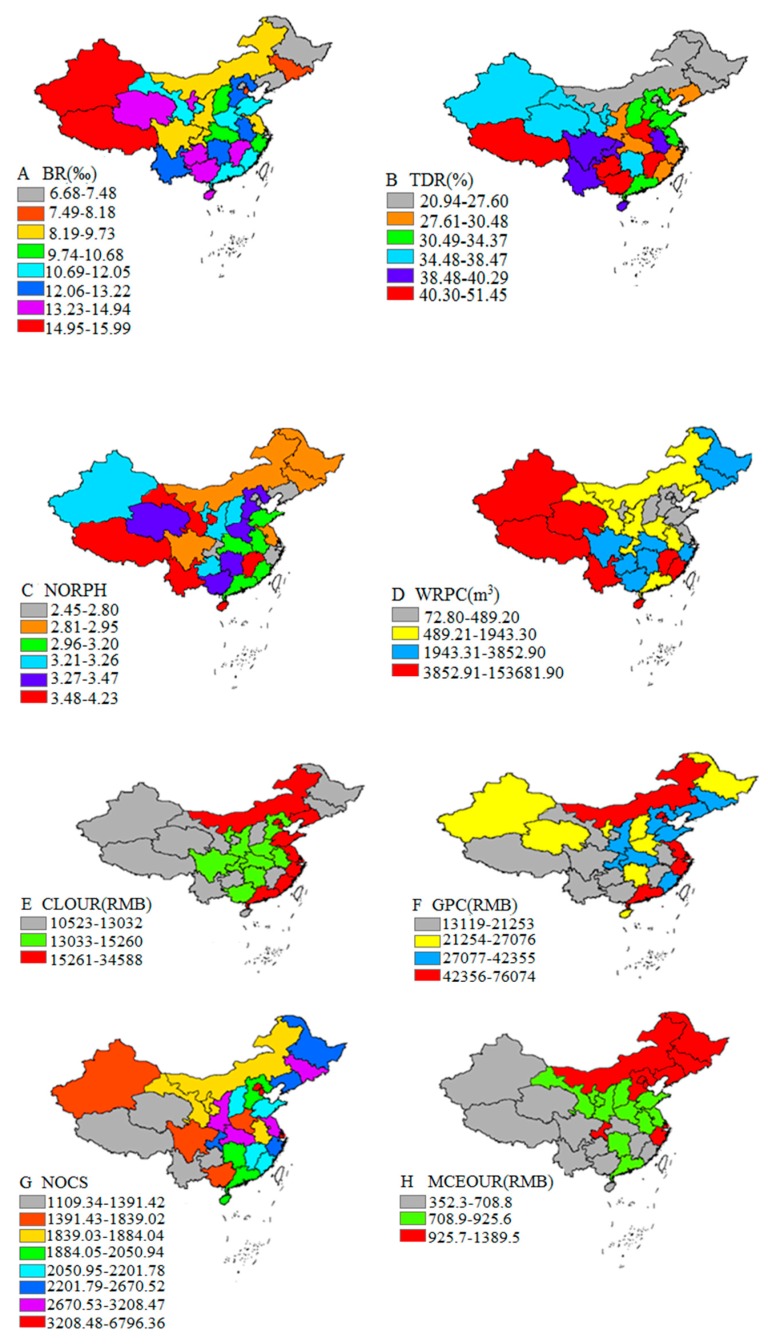

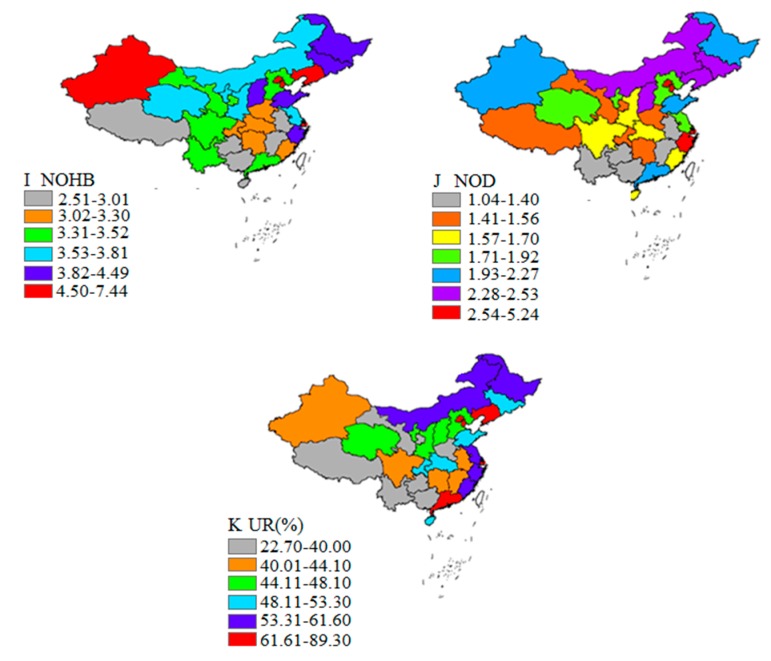
Spatial distribution map of social and environmental factors of LE in China in 2010. (**A**) Distribution of BR (Birth rate); (**B**) Distribution of TDR (Total dependency rate); (**C**) Distribution of NORPH (Number of residents per household); (**D**) Distribution of WRPC (Water resource per capita); (**E**) Distribution of CLOUR (Consumption level of urban residents); (**F**) Distribution of GPC (GDP per capita); (**G**) Distribution of NOCS (Number of college students per 100,000 persons; (**H**) Distribution of MCEOUR (Medical care expenditure of urban residents); (**I**) Distribution of NOHB (Number of hospital beds per 1000 persons); (**J**) Distribution of NOD (Number of doctors per 1000 persons); (**K**) Distribution of UR (Urbanization rate).

We also performed spatial maps of social and environmental factors to reveal their spatial characteristics (Figure 3). We can see that there exist certain spatial differences in BR, NORPH and TDR. And the three indicators show an upward trend from the east to the west, which were different from the spatial distribution of LE (Figure 3A–C). There were great spatial differences in the WRPC. For instance, the WRPC in Tibet reached the highest level (153,681.9 m^3^), while in Tianjin, it was only 72.8 m^3^. In addition, the WRPC increased from the north to the south in China (Figure 3D). NOHB and NOD showed a decline trend from the north to the south (Figure 3I–J). There existed big differences in the CLOUR, GPC, NOCS, MCEOUR and UR among all provinces in China, showing a downward trend from the east to the west (Figure 3E–H,K)

### 3.3. The Influence of Social and Environmental Factors on LE Based on Factor Detector

Based on the factor detector, we analyzed the importance of social and environmental factors on LE. Table 4 lists the *PD* value and its *p* value of each factor. The *PD* value of medical resources (NOHB, NOD) was not statistically significant (*p* > 0.05), which indicates that medical resources had no effect on LE at the provincial scale. However, the *PD* of most variables ranged from 0.45 to 0.65. In particular, the *PD* value of NOCS was 0.89, which indicates that NOCS can mainly explain the spatial stratified heterogeneity of LE in our study. Moreover, the *PD* values of WRPC and MCEOUR were less than 0.4, revealing that they had less influence on LE than other factors.

### 3.4. The Optimal Range of Factors for the Maximum LE Based on Risk Detector

**Figure 4:**
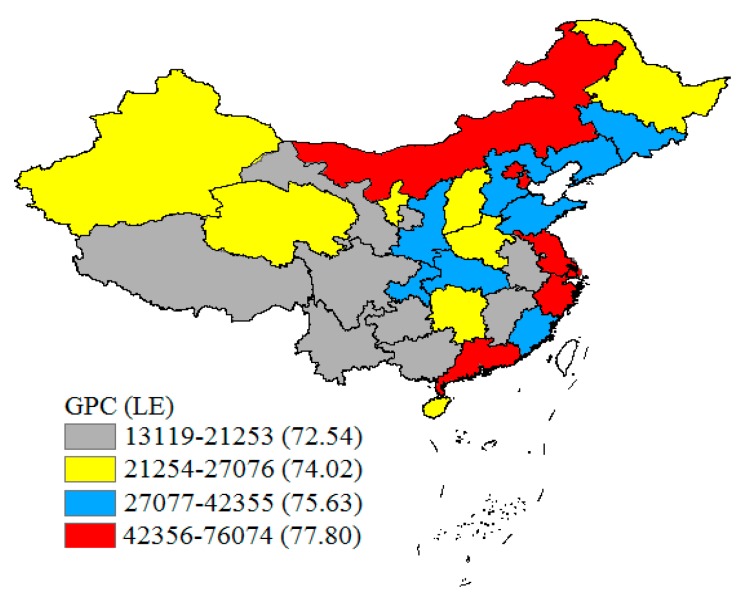
The subregions of the GPC and their LE.

**Figure 5:**
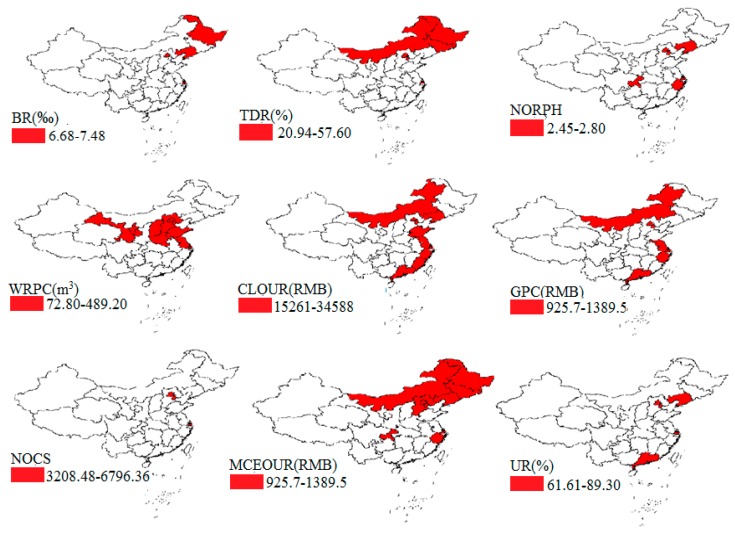
Distribution of main influencing area of each influencing factors.

The risk detector showed the average LE of each stratum of all factors and analyzed whether there was significant difference between each stratum. Taking GPC as an example: the relationship between GPC and LE is shown in Figure 4. With the increase of GPC, LE also increased. When the GPC ranged from 13,119 to 21,253 RMB, the average LE reached 72.54 years old. However, when the GPC ranged from 42,355 to 76,074 RMB, the average LE rose to 77.80 years old. The significance of the average LE differences between each stratum of GPC is shown in Table 5 (Y: significant, N: not significant). The fourth layer corresponds to the maximum range in Figure 4 (42,355 to 76,074 RMB), while the first layer corresponds to the minimum range in Figure 4 (13,119 to 21,253 RMB). We can see that the difference of average LE between the fourth layer and other layers of GPC was statistically significant in Table 5. Furthermore, the risk detector was able to analyze the quantitative relationship between social and environmental factors and LE. As could be seen in Table 6, the results show that the highest ranges of CLOUR, GPC, NOCS, MCEOUR and UR related to the highest level of LE, while the lowest ranges of BR, TDR, NORPH and WRPC corresponded to the highest level of LE. The optimal range of factors corresponds to the maximum value of the LE [36]. Therefore, we displayed the optimal range of factors in Table 6. It is worth noting that the areas with the highest LE value could be identified as the main influencing area for each significant social and environmental factor [34]. We show the results in Figure 5 with visualization technology.

### 3.5. Differences between the Impact of Different Factors on LE Based on the Ecological Detector

The significance of differences in *PD* values between two factors on LE was compared by the ecological detector (Table 7). The results show that the differences in *PD* values among most factors were not significant (Y: significant, N: not significant). The differences in *PD* values of NOCS and other factors were statistically significant, suggesting that NOCS had a great impact on LE. The *PD* value of WRPC was significantly different from that of BR and UR, respectively. Similarly, the difference in *PD* values of MCEOUR and UR was statistically significant. Combined with the results of the factor detector, this shows that the impact of WRPC and MCEOUR on LE was weak.

### 3.6. Interaction between Different Factors Based on the Interaction Detector

We used the interaction detector to reveal the interaction effect and types among the factors. As shown in Table 8, the *PD* value was ≥0.9 after NOCS interacted with other factors. Notably, the *PD* value of the interaction between NOCS and NORPH reached 0.98, which was closer to 1. The *PD* value of UR interacted with other factors was also high; for example, the *PD* value of UR that interacted with WRPC was 0.92. However, the *PD* value of the interaction between WRPC and MCEOUR was only about 0.5. We found that the interaction effect of any two factors was greater than the individual effect of a certain factor on LE. Even for the factors with a lower *PD* value, their *PD* value increased after the interaction. Moreover, the results show that all interaction types were bivariate-enhanced.

## 4. Discussion

LE is an important indicator for measuring health status [37]. To our knowledge, this is the first time that the relationship between LE and social and environmental factors is explored in China from spatial perspective using the Geographic Detector technique.

Many previous studies have shown that LE was mainly affected by economic factors [36,38,39]. For example, one study found that the main reason for the spatial distribution pattern of LE in China was the economy [40]. In contrast, we found that NOCS could mainly explain the spatial stratified heterogeneity of LE at the provincial scale combined with the results of factor detector and ecological detector, that is, the effect of NOCS on LE was significantly greater than that of the other factors. There is some possible explanation. Firstly, compared with other factors, people with higher education were related to good health awareness and more timely access to health care [15,41]; secondly, a higher education population could resist the adverse effects of negative aspects with better psychological quality [42]. At the same time, our study found that WRPC had little effect on LE. Some previous studies also found that this effect showed an upward trend from 2000 to 2010 [15]. Therefore, our government, especially in the eastern developed areas, should pay attention to the protection of the ecological environment while improving social economy. In addition, the impact of medical resources (NOHB, NOD) on LE was not statistically significant. Even though there were abundant medical resources in some areas, their actual efficiency might be very low due to poor infrastructure and low economic level. Therefore, they might not fully play a role, even had no effect on LE. However, the impact of MCEOUR on LE was also quite small, which further reveals the importance of improving the utilization of medical resources [43].

The results of the risk detector show that when the economic factors (GPC and CLOUR) and UR reached the maximum range, the average LE was also closer to the highest level. Because economic status played a role through its effects on people’s daily life, such as education, medical care, etc., the average LE would reach the maximum level with the GPC at the maximum value. The consumption level (CLOUR) was closely related to economic situation. When the consumption level reached the maximum value, people would purchase enough food to improve their health [44,45]. People who lived in the areas with highest UR would have the longest average LE because the high UR corresponded to the high economic conditions, medical and educational opportunities [10]; meanwhile, residents in rural areas also reported much higher rates of disability, injury and high blood pressure compared with urban residents, due to inequalities in education, health care and poverty [46]. Therefore, the Chinese government, especially in the central and western areas, should focus on the alleviation of poverty and urbanization to improve local LE. At the same time, when the BR and family living standard (TDR, NORPH) were in the minimum range, the average LE reached its maximum level. In general, the areas with the lowest BR were usually economically developed, such as Shanghai and Beijing. The social welfare in these areas was also higher than that of other areas [39]. Moreover, areas with a lowest BR tended to have the fewest number of families members (NORPH) and the lowest total dependency ratio (TDR). Therefore, they had the longest average LE.

Based on the interaction detector, we found that the *PD* value of any social environment factor interacted with NOCS was ≥0.9, indicating that education combined with other factors could significantly improve LE level. Therefore, the government, especially in Western China, should focus on improving the education as well as economic and medical conditions.

Our research shows the impact of social and environmental factors on life expectancy and their interaction [47]. We display the optimal range of factors for maximum LE and the main influencing area, which was meaningful for health policy development. Moreover, the selected variables covered multiple dimensions and the data on them were authoritative, which came from the national bureau of statistics. However, there are still some limitations in our study. Firstly, this study only focused on social and environmental factors, so there might be some factors influencing LE that were not included, such as air pollutants PM_2.5_, PM_10_, etc. However, we could not obtain the data of air pollution factors in each province in 2010. In addition, the data of LE and social and environmental factors used in this study were all from 2010, so they were insufficient in inferring a causal relationship. Moreover, the relationship between the dependent variable and independent variable was statistical and was not causality but the geographical detectors could filter out highly potential factors of LE for further confirmation, such as longitudinal studies [16]. In addition, this study was performed at the provincial level, which needs to be studied at a more precise scale in the future. Finally, the Geographical Detector could only explore the interaction effect between two factors and failed to further reveal the impact of multiple interactions on LE, which was also a key problem to be solved in the future.

## 5. Conclusions

In conclusion, there exist obvious spatial stratified heterogeneity of LE in China. Among the many social and environmental factors, NOCS could mainly explain the spatial stratified heterogeneity of LE. BR, TDR, NORPH, CLOUR, GPC and UR had less influence on LE, while WRPC and MCEOUR had the lowest influence on LE. Further study is needed to discover the actual causality between LE and these factors. When BR, TDR, NORPH and WRPC were at the minimum range, LE reached the highest level; conversely, LE reached the highest level with CLOUR, GPC, NOCS, MCEOUR and UR at the maximum range. In addition, the interaction of any two social and environmental factors on LE was stronger than the effect of a single factor. Our results provide political basis for the government to formulate economic and educational development, utilization of medical resources, environmental protection and population management policies to solve the regional inequality of LE in China.

## Figures and Tables

**Table 1:** Social and environmental factors in the study.

Categories 1	Categories 2	Variables	Abbreviation
Population structure	-	Birth rate	BR
Family living standard	Raising burden	Total dependency ratio	TDR
	Family size	Number of residents per household	NORPH
Environmental resources	-	Water resource per capita	WRPC
Economic development	Residents’ living	Consumption level of urban residents	CLOUR
	Economic level	GDP per capita	GPC
Education level	-	Number of college students per 100,000 persons	NOCS
Medical level	Medical expenditure	Medical care expenditure of urban residents	MCEOUR
	Medical resources	Number of hospital beds per 1000 persons	NOHB
		Number of doctors per 1000 persons	NOD
Other	-	Urbanization rate	UR

**Table 2:** Types of interaction between two covariates [34].

Description	Interaction
$PD(X_{1}\cap X_{2})<\mathrm{Min}(PD(X_{1}),PD(X_{2}))$	Weaken, nonlinear
$\mathrm{Min}(PD(X_{1}),PD(X_{2}))<PD(X_{1}\cap X_{2})<\mathrm{Max}(PD(X_{1})),PD(X_{2}))$	Weaken, univariate
$PD(X_{1}\cap X_{2})>\mathrm{Max}(PD(X_{1}),PD(X_{2}))$	Enhanced, bivariate
$PD(X_{1}\cap X_{2})=PD(X_{1})+PD(X_{2})$	Independent
$PD(X_{1}\cap X_{2})>PD(X_{1})+PD(X_{2})$	Enhance, nonlinear

**Table 3:** Descriptive analysis and discretization results of social and environmental factors.

Variables	Max	Min	Classification Interval
BR (‰)	15.99	6.68	8
TDR (%)	51.45	20.94	6
NORPH	4.23	2.45	6
WRPC (m^3^)	153,681.9	72.8	4
CLOUR(RMB)	34,588	10,523	3
GPC (RMB)	76,074	13,119	4
NOCS	6196.36	1109.34	8
MCEOUR (RMB)	1389.5	352.3	3
NOHB	7.44	2.51	6
NOD	5.24	1.04	7
UR (%)	89.3	22.7	6

**Table 4:** The result of factor detector about social and environmental factors of LE.

Variables	*PD*	*p*
BR	0.61	0.017
TDR	0.49	0.024
NORPH	0.57	0.004
WRPC	0.35	0.015
CLOUR	0.47	0.014
GPC	0.50	0.046
NOCS	0.89	0.000
MCEOUR	0.396	0.040
NOHB	0.33	0.621
NOD	0.52	0.647
UR	0.64	0.049

LE: Life expectancy; *PD*: *Power of Determinant*.

**Table 5:** Significance of average LE difference between different layers of GPC (GDP per capita).

Stratum	1	2	3	4
1				
2	N			
3	Y	N		
4	Y	Y	Y	

**Table 6:** The optimal range of social and environmental factors and maximum average LE.

Variables	Optimal Range	Maximum Average LE (years)
BR(‰)	6.68–7.48	78.20
TDR (%)	20.94–27.60	77.66
NORPH	2.45–2.80	78.19
WRPC (m^3^)	72.80–489.20	76.96
CLOUR(RMB)	15,261–34,588	77.32
GPC (RMB)	42,356–76,074	77.80
NOCS	3208–6796	79.78
MCEOUR (RMB)	925.70–1389.50	77.07
UR (%)	61.61–89.30	78.44

**Table 7:** Statistical significance of the differences in PD values among different factors.

Impact Factors	BR	TDR	NORPH	WRPC	CLOUR	GPC	NOCS	MCEOUR	UR
BR									
TDR	N								
NORPH	N	N							
WRPC	Y	N	N						
CLOUR	N	N	N	N					
GPC	N	N	N	N	N				
NOCS	Y	Y	Y	Y	Y	Y			
MCEOUR	N	N	N	N	N	N	Y		
UR	N	N	N	Y	N	N	Y	Y	

**Table 8:** *PD* values for interactions between factors on the LE.

Impact Factors	BR	TDR	NORPH	WRPC	CLOUR	GPC	NOCS	MCEOUR	UR
BR	0.61								
TDR	0.86	0.49							
NORPH	0.85	0.75	0.57						
WRPC	0.85	0.73	0.79	0.35					
CLOUR	0.78	0.69	0.71	0.64	0.47				
GPC	0.84	0.63	0.79	0.69	0.65	0.50			
NOCS	0.97	0.96	0.98	0.95	0.96	0.94	0.89		
MCEOUR	0.68	0.60	0.65	0.52	0.66	0.59	0.90	0.40	
UR	0.89	0.81	0.85	0.92	0.80	0.74	0.95	0.78	0.64

## References

[1] Gou X. (2013). Quantitative analysis of life expectancy differences and influencing factors in countries around the world. J. Nanjing Coll. Popul. Program. Manag..

[2] The World Bank Database. Https://data.worldbank.org.cn.

[3] Guo Y. (2018). Analysis of spatio-temporal changes in life expectancy and its influencing factors in China. Chin. J. Health Policy.

[4] Yu T. (2017). Analysis on the Influencing Factor and Prediction of Life Expectancy. Master’s Thesis.

[5] Manuel D.G., Perez R., Sanmartin C., Taljaard M., Hennessy D., Wilson K., Tanuseputro P., Manson H., Bennett C., Tuna M. (2016). Measuring Burden of Unhealthy Behaviours Using a Multivariable Predictive Approach: Life Expectancy Lost in Canada Attributable to Smoking, Alcohol, Physical Inactivity, and Diet. PLoS Med..

[6] Song X., Chen G., Zheng X. (2010). Chinese Life Expectancy and Policy Implications. Procedia—Soc. Behav. Sci..

[7] Gilligan A.M., Skrepnek G.H. (2015). Determinants of life expectancy in the Eastern Mediterranean Region. Health Policy Plan.

[8] Wilkinson R.G. (2002). Unhealthy Societies: The Afflictions of Inequality.

[9] Rodgers G.B. (1979). Income and inequality as determinants of mortality: An international cross-section analysis. Popul. Stud..

[10] Lu X., Chen X. (2007). Factors on mean lifespan of each province in China. Yunan Geogr. Environ. Res..

[11] Yang Z., Liu H., Wang X. (2017). Spatio-temporal variations of population health distribution in China and its influencing factors. World Reg. Stud..

[12] Meara E.R., Richards S., Cutler D.M. (2008). The gap gets bigger: Changes in mortality and life expectancy, by education, 1981–2000. Health Affairs.

[13] Hao Y. (2003). A stepwise regression analysis of comprehensive environmental factors affecting life expectancy in Shanghai. Economist.

[14] Dong L., Li C. (2009). Relationship between life expectancy, GNP and education level. Stat. Decis..

[15] Jiang J., Luo L., Xu P., Wang P. (2018). How does social development influence life expectancy? A geographically weighted regression analysis in China. Public Health.

[16] Wang J.F., Li X.H., Christakos G., Liao Y.L., Zhang T., Gu X., Zheng X.Y. (2010). Geographical Detectors-Based Health Risk Assessment and its Application in the Neural Tube Defects Study of the Heshun Region, China. Int. J. Geogr. Inf. Sci..

[17] Zhou C., Chen J., Wang S. (2018). Examining the effects of socioeconomic development on fine particulate matter (PM2.5) in China’s cities using spatial regression and the geographical detector technique. Sci. Total Environ..

[18] Wang J.F., Zhang T.L., Fu B.J. (2016). A measure of spatial stratified heterogeneity. Ecol. Indic..

[19] Ju H., Zhang Z., Zuo L., Wang J., Zhang S., Wang X., Zhao X. (2016). Driving forces and their interactions of built-up land expansion based on the geographical detector—A case study of Beijing, China. Int. J. Geogr. Inf. Sci..

[20] Ren Y., Deng L., Zuo S., Luo Y., Shao G., Wei X., Hua L., Yang Y. (2014). Geographical modeling of spatial interaction between human activity and forest connectivity in an urban landscape of southeast China. Landscape Ecol..

[21] Tan J., Zhang P., Lo K., Li J., Liu S. (2016). The Urban Transition Performance of Resource-Based Cities in Northeast China. Sustainability.

[22] Lou C.R., Liu H.Y., Li Y.F., Li Y.L. (2016). Socioeconomic Drivers of PM2.5 in the Accumulation Phase of Air Pollution Episodes in the Yangtze River Delta of China. Int. J. Environ. Res. Public Health.

[23] Todorova Y., Lincheva S., Yotinov I., Topalova Y. (2016). Contamination and Ecological Risk Assessment of Long-Term Polluted Sediments with Heavy Metals in Small Hydropower Cascade. Water Resour. Manag..

[24] Hu Y., Wang J., Li X., Ren D., Zhu J. (2011). Geographical detector-based risk assessment of the under-five mortality in the 2008 Wenchuan earthquake, China. PLoS ONE.

[25] National Bureau of statistics of the People’s Republic of China (2012). China Statistical Yearbook 2012.

[26] National Bureau of Statistics of the People’s Republic of China (2011). China Population & Employment Statistics Yearbook 2011.

[27] National Health and Family Planning Commission of PRC (2011). China Health Statistical Yearbook 2011.

[28] National Bureau of statistics of the People’s Republic of China (2011). China Statistical Yearbook 2011.

[29] National Bureau of statistics of the People’s Republic of China (2014). China Statistical Yearbook 2014.

[30] Sridharan S., Tunstall H., Lawder R., Mitchell R. (2007). An exploratory spatial data analysis approach to understanding the relationship between deprivation and mortality in Scotland. Soc. Sci. Med..

[31] Wang S., Luo K. (2018). Life expectancy impacts due to heating energy utilization in China: Distribution, relations, and policy implications. Sci. Total Environ..

[32] Anselin L. (1995). Local Indicators of Spatial Assocation-LISA. Geogr. Anal..

[33] Wang J.F., Wang Y., Zhang J., Christakos G., Sun J.L., Liu X., Lu L., Fu X.Q., Shi Y.Q., Li X.M. (2013). Spatiotemporal transmission and determinants of typhoid and paratyphoid fever in Hongta District, Yunnan Province, China. PLoS Negl. Trop. Dis..

[34] Bai L., Jiang L., Yang D.-Y., Liu Y.-B. (2019). Quantifying the spatial heterogeneity influences of natural and socioeconomic factors and their interactions on air pollution using the geographical detector method: A case study of the Yangtze River Economic Belt, China. J. Clean. Prod..

[35] Cao F., Ge Y., Wang J.-F. (2013). Optimal discretization for geographical detectors-based risk assessment. GISci. Remote Sens..

[36] Zha X., Tian Y., Gao X., Wang W., Yu C. (2019). Quantitatively evaluate the environmental impact factors of the life expectancy in Tibet, China. Environ. Geochem. Health.

[37] Wang L., Binggan W., Li Y., Li H., Zhang F., Rosenberg M., Yang L., Huang J., Krafft T., Wang W. (2014). A study of air pollutants influencing life expectancy and longevity from spatial perspective in China. Sci. Total Environ..

[38] Lin R.-T., Chen Y.-M., Chien L.C., Chan C.C. (2012). Political and social determinants of life expectancy in less developed countries: A longitudinal study. BMC Public Health.

[39] Duque A.M., Peixoto M.V., Lima S.V., Goes M.A.O., Santos A.D., Araújo K.C.G.M., Nunes M.A.P. (2018). Analysis of the relationship between life expectancy and social determinants in a north-eastern region of Brazil, 2010–2017. Geospat. Health.

[40] Wang S., Luo K., Liu Y. (2015). Spatio-temporal distribution of human lifespan in China. Sci. Rep..

[41] Chan M.F. (2015). The impact of health care resources, socioeconomic status, and demographics on life expectancy: A cross-country study in three Southeast Asian countries. Asia Pac. J. Public Health.

[42] Shkolnikov V.M., Andreev E.M., Jasilionis D. (2006). The changing relation between education and life expectancy in central and eastern Europe in the 1990s. J. Epidemiol. Community Health.

[43] Ming Y., Dong Z. (2010). Life expectancy of China’ s population analysis of the impact of factors. Theory Res..

[44] Anand S., Ravallion M. (1993). Human Development in Poor Countries: On the Role of Private Incomes and Public Services. J. Econom. Perspect..

[45] Zheng C. (2010). Analysis of regional differences in life expectancy and economic and social factors. China Collect. Economy.

[46] Singh G.K., Siahpush M. (2014). Widening rural–urban disparities in life expectancy, US, 1969–2009. Am. J. Prev. Med..

[47] Huang J., Wang J., Bo Y., Xu C., Hu M., Huang D. (2014). Identification of health risks of hand, foot and mouth disease in China using the geographical detector technique. Int. J. Environ. Res. Public Health.

